# Wear state identification of reciprocating sliding friction Pairs with frictional vibration

**DOI:** 10.1371/journal.pone.0329782

**Published:** 2025-08-14

**Authors:** Haijie Yu, Haijun Wei

**Affiliations:** 1 Yazhou Bay Innovation Institute, International Navigation College, Hainan Tropical Ocean University, Sanya, China; 2 Merchant Marine College, Shanghai Maritime University, Haigang Av. Shanghai, China; China University of Mining and Technology, CHINA

## Abstract

Real-time monitoring of the wear state of reciprocating sliding friction pairs has long been a challenging issue. To address this problem, this paper innovatively proposes a new method of constructing feature vectors based on the fractal parameters of frictional vibration signals and employing a nonlinear support vector machine to identify different wear states. Three typical wear states, namely running-in wear, normal wear, and severe wear, were designed by adjusting the amount of lubricating oil and distinguished by variations in the friction coefficient. Unlike conventional time-frequency or statistical features, our approach uniquely employs multifractal spectrum parameters to characterize wear states. The research results demonstrate that this method achieves recognition accuracies exceeding 90% for all three wear states in 10-fold cross-validation, indicating the effectiveness of the nonlinear support vector machine in realizing the recognition of different wear states of reciprocating sliding friction pairs. This achievement not only provides a new technical approach for online monitoring of wear states but also offers a valuable reference for the application of nonlinear signal analysis in other fields.

## 1. Introduction

In the process of friction and wear, the reciprocating sliding friction pair will produce a lot of friction characteristics, such as frictional vibration, friction torque, friction coefficient, wear particles, wear surface morphology, etc. It is very difficult to obtain real-time tribological information such as friction torque, friction coefficient and wear surface morphology during wear of friction pairs [1–3]. At the same time, the collection and analysis of wear particles is very complex and time-consuming. Therefore, it is not an effective method to describe and distinguish wear states by using friction characteristics such as wear surface, wear particles, friction torque and friction coefficient. In contrast, frictional vibration signals during wear are easy to obtain in real time.

The frictional vibration signal possesses multifractal characteristics, and scholars conducted numerous investigations regarding it. In literature [4], the fractal characteristics of frictional vibration signals are analyzed by correlation dimension, and it is pointed out that the fractal dimension of friction and vibration signals in running-in wear process gradually increases, while the correlation dimension of friction and vibration signals in dry friction process gradually decreases. In reference [5], a multifractal analysis was made on the frictional vibration signals in the running-in process, and it was pointed out that the multifractal spectrum parameters of frictional vibration could be used to characterize the running-in process. In literature [6], the empirical mode decomposition method and the overall empirical mode decomposition method were respectively used to denoise the measured vibration signals, and the improved multifractal method was used to analyze the characteristics of frictional vibration signals, and good results were obtained. Moreover, a novel method has been put forward for extracting the fractal dimension features of the wear state of a deep-hole drill bit. This method employs the binary wavelet function as the scale and is based on wavelet fractal dimension [7]. The dynamic relationship between the friction coefficient and fractal parameters is derived from a microscopic contact perspective, and the wear periods are determined based on the dynamic changes of fractal parameters [8]. In reference [9], a relationship between the change of the generalized Hurst exponent and tool wear was established by using the multifractal trend fluctuation analysis. The multifractal detrended fluctuation analysis method was used to conduct a multifractal feature analysis on the cavitation vibration signals of the water turbine [10]. By using the variational mode decomposition (VMD) method, the grinding chatter signal was decomposed into intrinsic mode functions (IMFs) covering different frequency bands. The multifractal spectrum widths of each intrinsic mode function were calculated, and it was found that the intrinsic mode function IMF-4 of rail grinding has the most obvious multifractal characteristics [11]. However, recent works [7–11] only pointed out that the frictional vibration signal has multifractal characteristics, and did not propose a wear state identification method of reciprocating sliding friction pairs based on these characteristics.

Support vector Machine (SVM) is an intelligent optimization algorithm based on the structural risk minimization principle, which is based on the statistical theory. It can solve both linear optimization problems and nonlinear optimization problems, and is widely used in fault diagnosis and pattern recognition [12–15]. In this paper, feature vectors are constructed based on fractal parameters of frictional vibration signals, and three wear states of running-in wear, normal wear and severe wear are identified by nonlinear support vector machine. The novelty of this work lies in the first-time integration of fractal parameters derived from frictional vibration signals with a nonlinear SVM classifier for wear state identification in reciprocating sliding friction pairs. Unlike existing studies that rely on traditional features (e.g., time-domain statistics or frequency spectra), we leverage multifractal spectrum parameters to capture the nonlinear dynamics of wear states. This approach is validated across five distinct operating conditions, demonstrating robustness to variations in loading force and reciprocating frequency.

## 2. Method

**Fig 1:**
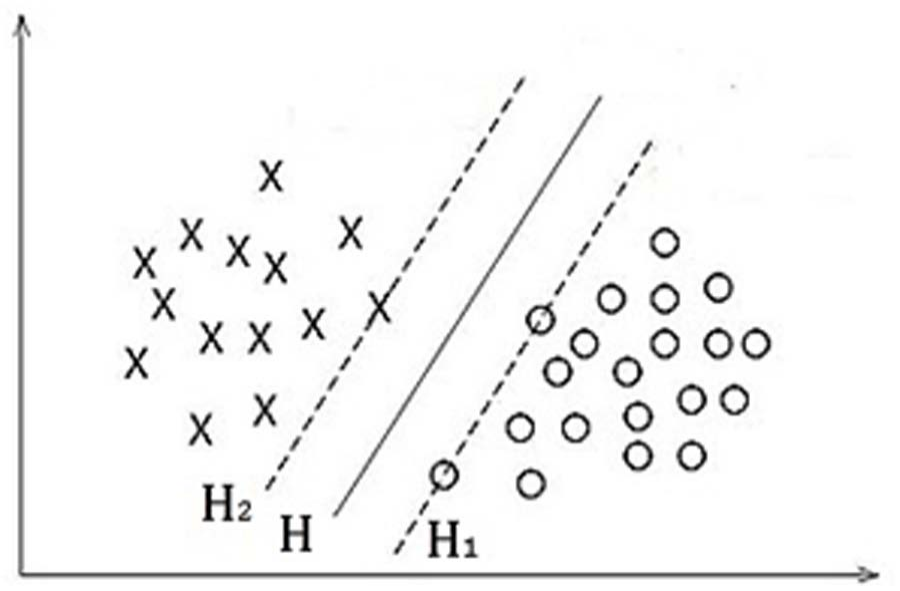
Linearly separable optimal classification line.

The SVM method is generalized from finding the optimal classification line in the problem of linear separability. Its main idea is shown in Fig 1. H is the classification line of two kinds of samples, $H_{1}$ and $H_{2}$ are the lines parallel to H through the sample point and the closest distance to H, and the distance between $H_{1}$ and $H_{2}$ is called the classification interval. The optimal classification line to be obtained should be able to separate the two classes completely and normally, and at the same time make the classification interval maximum [16].

The classification surface can be represented by the equation wx+b=0, where w is the normal vector and b is the offset. According to the maximum classification interval of the optimal classification surface, the sample points will not fall on the classification line defined by the equation wx+b=0. Therefore, the module of w can be limited to the reciprocal distance from the nearest point to the hyperplane through the following constraints [17]:


$$\begin{array}{c} \min_{x}|wx+b|=1 \end{array}$$ (1)

For the training set$\left(x_{1},y_{1}\right),\left(x_{2},y_{2}\right),\cdots ,\left(x_{n},y_{n}\right),x_{i}\in R_{N},y\in \left\{-1,1\right\}$, the optimal classification line has the following constraints:


$$\begin{array}{c} y_{i}\left(\left(wx_{i}\right)+b\right)\geq 1,i=1,2,\cdots ,n \end{array}$$ (2)

Distance from x to the hyperplane (w,b) is:


$$\begin{array}{c} d(w,b;x)=\frac{\left|(wx)+b\right|}{\left||w|\right|} \end{array}$$ (3)

The classification interval can be expressed as:


$$\begin{array}{c} \rho (w,b)=\frac{2}{\left||w|\right|} \end{array}$$ (4)

The problem of finding the optimal hyperplane is transformed into a quadratic optimization problem solving the following constraints:


$$\begin{array}{c} min\phi (w)=\frac{1}{2}{\left||w|\right|}^{2} \end{array}$$ (5)

From optimization theory, the above problem is transformed into an unconstrained optimization problem by Lagrange multiplier method:


$$\begin{array}{c} \begin{array}{c} \min_{w,b}L(w,b,\alpha )=\frac{1}{2}{\left||w|\right|}^{2}-\sum_{i=1}^{n}\alpha_{i}\left(\left(wx_{i}+b\right)y_{i}-1\right)\\ s.t.\;y_{i}(wx+b)\geq 1,i=1,2,\cdots ,n \end{array} \end{array}$$ (6)

Where: $\alpha_{i}\geq 0$ is the Lagrange multiplier.

According to the unconstrained optimization theory, the necessary conditions for L(w,b,α) to take the extreme value are:


$$\begin{array}{c} \left\{ \begin{array}{c} \frac{\partial L}{\partial b}=0\\ \frac{\partial L}{\partial w}=0 \end{array}\right.\; \end{array}$$ (7)

Thus, can be obtained


$$\begin{array}{c} \left\{ \begin{array}{c} \sum_{i=1}^{n}\alpha_{i}y_{i}=0\\ w=\sum_{i=1}^{n}\alpha_{i}x_{i}y_{i} \end{array}\right.\; \end{array}$$ (8)

The dual form of the optimization problem is obtained by substituting equation (8) into equation (6):


$$\begin{array}{c} \left\{ \begin{array}{c} \max_{\alpha }W(\alpha )=\max_{\alpha }\sum_{i=1}^{n}\alpha_{i}-\frac{1}{2}\sum_{i=1}^{n}\sum_{j=1}^{n}\alpha_{i}\alpha_{j}y_{i}y_{j}\left(x_{i}x_{i}\right)\\ s.t.\alpha_{i}\geq 0,i=1,2,\cdots ,l\;\\ \sum_{i=1}^{l}\alpha_{i}y_{i}=0 \end{array}\right.\; \end{array}$$ (9)

The solution of the equation is:


$$\begin{array}{c} \alpha^{*}=argmin\frac{1}{2}\sum_{i=1}^{n}\sum_{j=1}^{n}\alpha_{i}\alpha_{j}y_{i}y_{j}\left(x_{i}x_{i}\right)-\sum_{i=1}^{l}\alpha_{i} \end{array}$$ (10)

Thus, the optimal hyperplane can be obtained as:


$$\begin{array}{c} w^{*}=\sum_{i=1}^{l}{\alpha_{i}}^{*}x_{i}y_{i} \end{array}$$ (11)

By setting the relaxation variable ξ and the penalty factor c, equation (11) is generalized to the generalized optimal hyperplane problem:


$$\begin{array}{c} \left\{ \begin{array}{c} \min\Phi (w,\xi )=\frac{1}{2}{\left||w|\right|}^{2}+c\sum_{i=1}^{n}\xi_{i}\\ s.t.\;y_{i}\left(wx_{i}+b^{*}\right)\geq 1-\xi_{i},\;i=1,2,\cdots ,n \end{array}\right.\; \end{array}$$ (12)

Where: The relaxation variable ξ represents the measure of the error classification; A smaller penalty factor c means a smaller penalty for misclassification. The generalized optimal hyperplane is determined by appropriately selecting the maximum classification interval with the minimum number of error classification samples, and the minimum value of Φ(w,ξ) is obtained.

**Fig 2:**
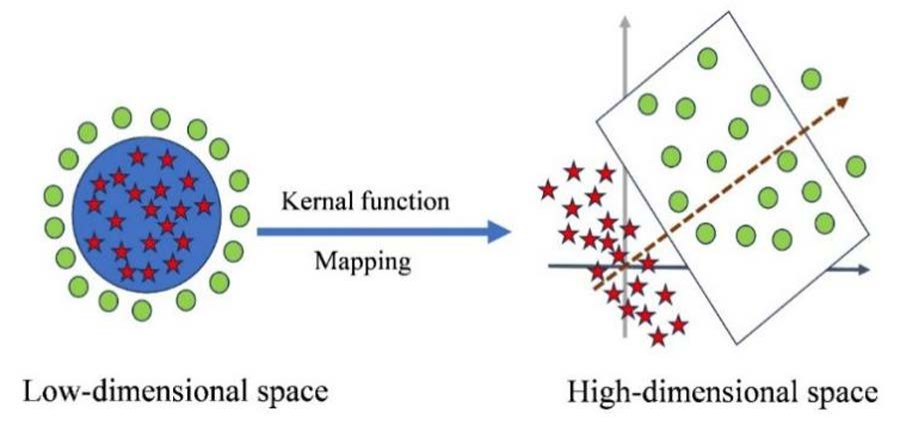
Linearly indivisible maps to linearly divisible space.

When the data set is linearly non-separable, SVM method can map the original data into a high-dimensional space with suitable kernel functions. As shown in Fig 2, in the low-dimensional space, the data is linearly indivisible, and the linear indivisible data is mapped to the high-dimensional space through the kernel function, so that the data is linearly divisible in the high-dimensional space [16].

According to the above ideas, by setting the appropriate mapping Φ:x→Φ(x), the original data is mapped to the high-dimensional feature space, where the possibility of linear divisible data is greatly increased, and then using the linear divisible solution method, the optimization problem shown in equation (13) is solved:


$$\begin{array}{c} \left\{ \begin{array}{c} \alpha^{*}=argmin\frac{1}{2}\sum_{i=1}^{n}\sum_{j=1}^{n}\alpha_{i}\alpha_{j}y_{i}y_{j}\left(\Phi \left(x_{i}\right)\cdot \Phi \left(x_{j}\right)\right)-\sum_{i=1}^{l}\alpha_{i}\\ s.t.{0\leq \alpha }_{i}\leq c,i=1,2,\cdots ,l\;\\ \sum_{i=1}^{l}\alpha_{i}y_{i}=0 \end{array}\right.\; \end{array}$$ (13)

## 3. Experiment

### 3.1 Experimental facility

**Fig 3:**
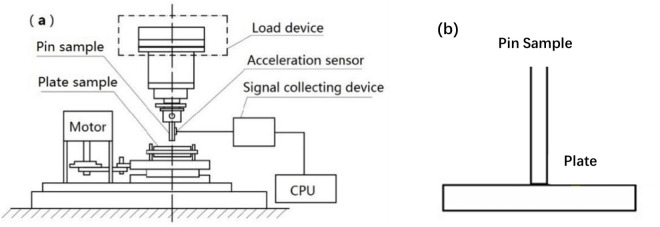
Schematic diagram of tester. (a) experiment device; and (b) tribological pairs.

The friction-wear test was carried out on the friction and wear test machine. As shown in Fig 3, the pin sample was fixed on the upper part of the test bench, the plate sample was placed in the lower tray, the three-axis acceleration sensor was attached to the side of the fixture dedicated to the pin sample, the servo motor drove the block sample to reciprocate through the eccentric mechanism, and the loading device at the top controlled the loading force. The device has built-in sensors to measure the normal loading force and friction force. The test conditions such as reciprocating frequency, stroke displacement and load force can be set through the computer software provided with the equipment, and the information such as friction force, friction coefficient and stroke displacement can be recorded in real time during the test. To acquire the vibration signals, a triaxial acceleration sensor (model 356B17ICP, manufactured by PCB PIEZOTRONICS Company) was attached to the pin specimen. This sensor features a measurement range of ±5g and a sensitivity of 1000mv/g. The data acquisition process was executed by utilizing a data acquisition system (VibPilot, provided by m + p international).

### 3.2 Experimental method

The experiment was divided into two groups. The first group was the continuous running-in process experiment under oil immersion condition, which experienced running-in wear state and normal wear state. The two wear states were divided by observing the change trend of friction coefficient during the experiment. The other group is the severe wear experiment produced in the condition of no lubricating oil.

#### 3.2.1 Running-in wear test.

The running-in wear process is characterized by many influencing factors and poor repeatability. To study the generalization ability of the identification method and consider the required running-in time, tests under five working conditions were selected. The working conditions are shown in Table 1.

**Table 1:** Running-in wear test operating parameters.

Test number	Loading force/N	Frequency/Hz	Wear state	Test time/min
1.1	50	0.5	Running-in→Normal	90
1.2	60	0.5	Running-in→Normal	90
1.3	70	0.5	Running-in→Normal	90
1.4	50	1	Running-in→Normal	90
1.5	50	2	Running-in→Normal	90

The relative position of each group of test pin and plate was different. At the beginning of each test, a new upper sample was replaced. The reciprocating sliding stroke is 16 mm. The pin sample is a cylinder with a section diameter of 6 mm and a length of 35 mm. It is made of gray cast iron with carbon, sulfur, silicon, iron, phosphorus, manganese, etc. The surface hardness is 249 HV, and the initial surface roughness $S_{a}$=0.856 μm. The plate sample length is 43.3 mm, the width is 35 mm, and the thickness is 2.9 mm. It is made of alloy cast iron of the same material as the cylinder liner of marine diesel engine. The element composition is carbon, silicon, iron, phosphorus, manganese, etc. The surface hardness is 341 HV, the metallographic structure is sostenite, graphite, pearlite, and the initial surface roughness $S_{a}$=0.146 μm.

**Fig 4:**
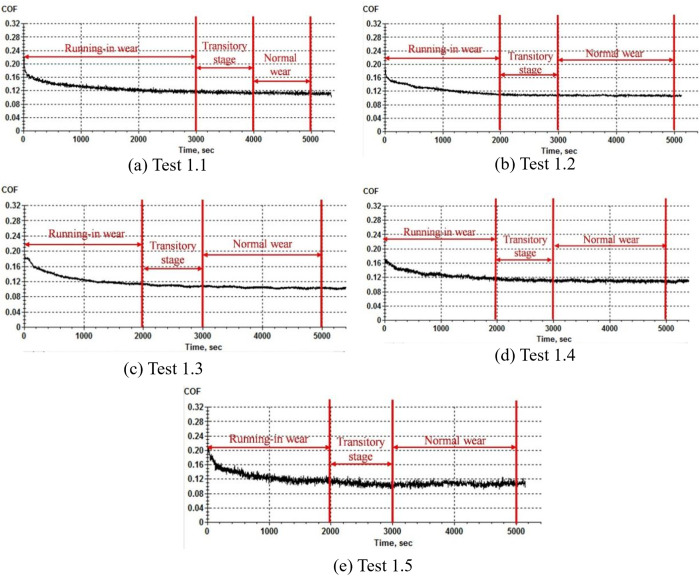
Diagram of friction coefficient variation in running-in wear test.

Fig 4 shows the variation of friction coefficient during running-in under various test conditions. As can be seen from the figure, although the initial friction coefficient is different under different working conditions, the friction coefficient gradually decreases from a large value at the beginning with the progress of the test and finally stabilates around 0.1, indicating that the loading force and reciprocating frequency have little influence on the friction coefficient in the stable state after running-in. When the friction coefficient decreases continuously, the friction pair experiences running-in wear. When the friction coefficient is stable at about 0.1, the macroscopic lubrication state of the surface friction pair is fluid lubrication, and the wear state is normal wear. As can be seen from Fig 4a, test 1.1 enters the normal wear state at the end of the running-in process around 3500 s; As can be seen from Fig 4b, test 1.2 enters the normal wear state at the end of the running-in process around the 2500s. As can be seen from Fig 4c, test 1.3 enters the normal wear state at the end of the running-in process around the 2300 s. As can be seen from Fig 4d, test 1.4 enters the normal wear state at the end of the running-in process around the 2500 s. As can be seen from Fig 4e, test 1.5 enters the normal wear state at the end of the running-in process around the 2500 s. To better distinguish the vibration signal samples under different wear states, a transition stage is added between running-in wear and normal wear, and the vibration signal samples collected in this stage are uncertain samples.

#### 3.2.2 Severe wear test.

There are many reasons for the severe wear of reciprocating sliding friction pairs, such as the mismatch between the cylinder oil and the fuel of the two-stroke diesel engine, the insufficient oil injection rate of the cylinder oil, poor combustion, cylinder oil deterioration, etc. The above internal mechanisms first cause poor lubrication on the surface of the friction pair, thereby reducing the service life of the piston ring and eventually leading to the loss of its function. Therefore, this paper simulates the severe wear caused by the above reasons by removing the lubricating oil on the surface of the friction pair. After the end of each group of running-in wear test, the severe wear test was carried out. The lubricating oil on the surface of the friction pair was absorbed with non-woven paper and cleaned with gasoline. The test condition corresponds to the running-in wear test one by one, and the working condition parameters are shown in Table 2.

**Table 2:** Severe wear test operating parameters.

Test number	Loading force/N	Frequency/Hz	Wear state	Test time/min
2.1	50	0.5	Severe	45
2.2	60	0.5	Severe	45
2.3	70	0.5	Severe	45
2.4	50	1	Severe	45
2.5	50	2	Severe	45

**Fig 5:**
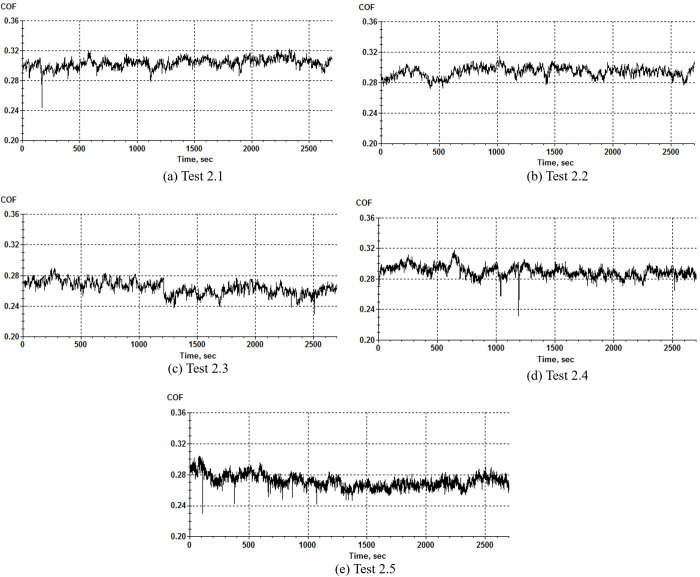
Diagram of friction coefficient variation in severe wear test.

Fig 5 shows the variation of friction coefficient of severe wear test under various test conditions. As can be seen from the figure, the friction coefficient is large and has obvious fluctuations under severe wear state. In the range of experimental conditions, the friction coefficient decreases with the increase of loading force and reciprocating frequency.

## 4. Multifractal characteristics analysis of frictional vibration signals

### 4.1 Multifractal spectrum of frictional vibration signals

**Fig 6:**
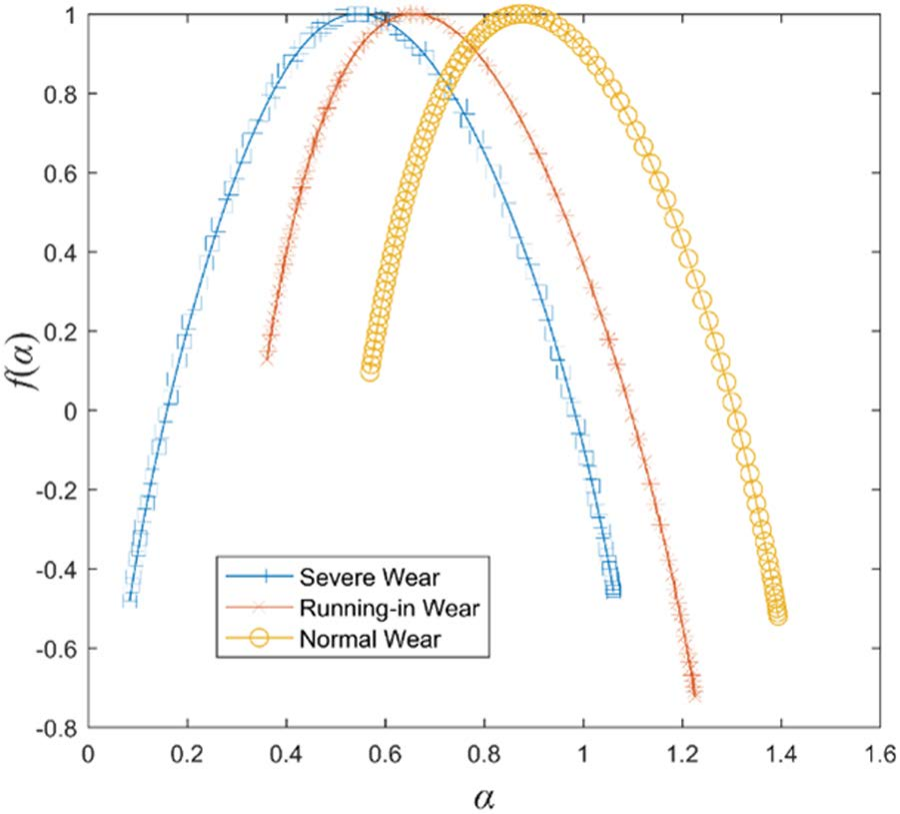
Multifractal spectrum of frictional vibration under different wear states.

Firstly, the measured vibration signals were denoised by the method mentioned in reference [18]. Then, the multifractal spectra corresponding to the frictional vibration signals under the three wear states were calculated by the multifractal detrended fluctuation analysis method [19,20], and the results are shown in Fig 6. A larger singularity index α corresponds to a smaller scale wave function, and conversely, a smaller singularity index α corresponds to a larger scale wave function [21]. By comparing the multifractal spectrum of the frictional vibration signals under the three wear states, it is found that the value of the singular index of the frictional vibration signals under the normal wear state is larger, corresponding to the smaller scale wave function, indicating that the frictional vibration signals are weak under the normal wear state. Under severe wear state, the singular index value of frictional vibration signal is small, corresponding to a larger scale wave function, indicating that the frictional vibration is strong under severe wear state. The value range of the singular index, that is, the spectrum width, corresponds to the distribution of the wave function, and the wider the spectrum corresponds to the wider the distribution of the wave function, which means that the signal fluctuation range is wider, that is, the signal is coarser. The multifractal spectrum width of frictional vibration signals in three states is compared, and it is found that the multifractal spectrum width of frictional vibration signals in normal wear state is the smallest, which indicates that the fluctuation range of frictional vibration signals in normal wear state is small. The multifractal spectrum width of the frictional vibration signal under severe wear state is larger, which indicates that the fluctuation range of the frictional vibration signal is larger under severe wear state.

### 4.2 Characteristic parameter analysis

**Fig 7:**
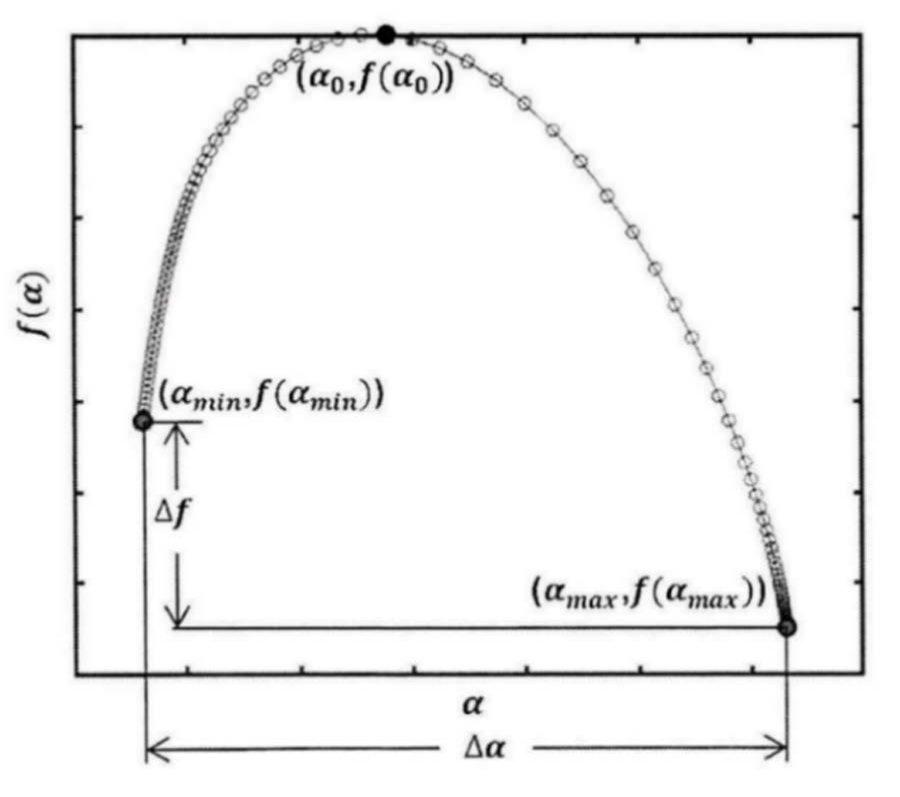
Schematic diagram of multifractal spectrum parameters.

It can be seen from the above analysis that the fractal spectrum of frictional vibration signals under different wear states has different shape and position characteristics. To obtain the classification model, the input parameters are the sample eigenvector parameter. Therefore, the eigenvector parameters representing the fractal characteristics are selected from the singularity index α and the fractal dimension f(α) which constitute the multifractal spectrum. As shown in Fig 7, they are $\alpha_{\min}$, $\alpha_{\max}$, $f(\alpha_{\min})$, $f(\alpha_{\max})$, Δα, Δf, $\alpha_{0}$ respectively.

**Fig 8:**
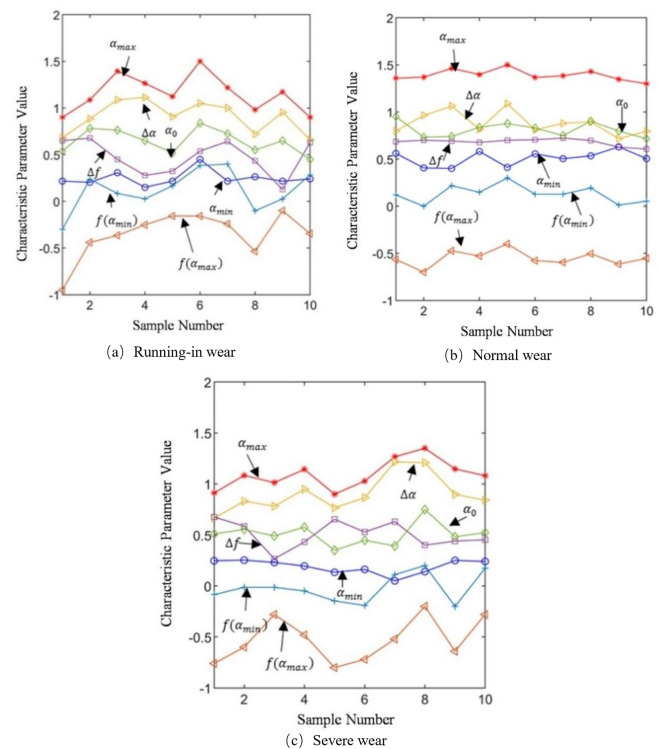
Stability of spectral parameters.

For the purpose of exploring the stability and sensitivity of multifractal spectrum parameters, ten groups of samples, each with duration 2 s under different wear conditions at 50 N and 0.5 Hz, were randomly selected. The value distribution of the multifractal spectrum parameters is shown in Fig 8. As can be seen from the figure, the value distribution of sample characteristic parameters in the normal wear state is relatively stable. In contrast, the value range of the sample characteristic parameters in running-in wear state is relatively large, while the value range of sample characteristic parameters in the severe wear state lies in the middle. This is consistent with the range characteristics of the friction coefficient changes in each state, thereby indicating that multifractal spectrum parameters can be used to characterize different wear states.

**Fig 9:**
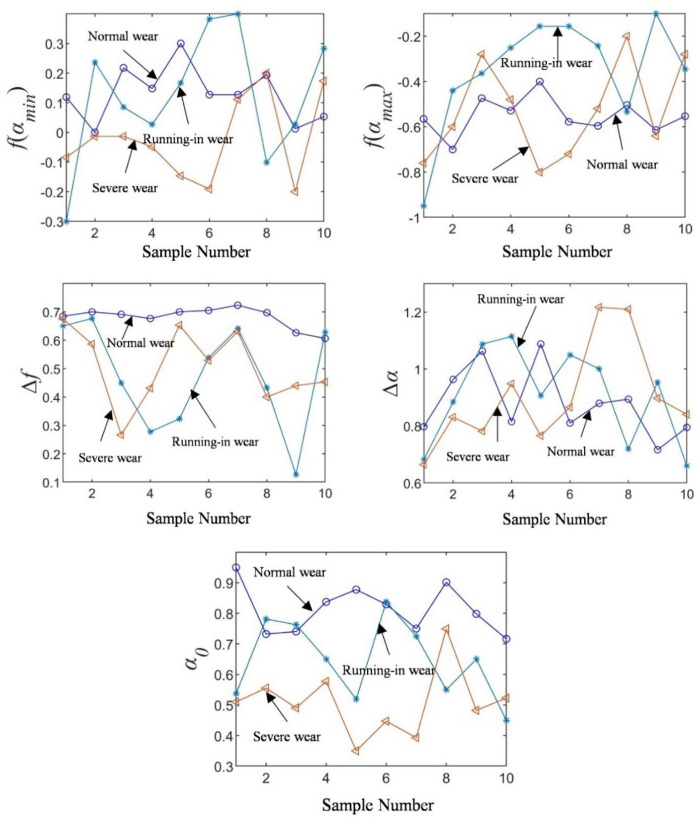
Sensitivity of spectral parameter.

Fig 9 shows the sensitivity of different multifractal spectrum parameters to wear states, which can measure the feasibility of interval division of data for different states. The seven groups of characteristic parameters have certain state aliasing for different wear states, indicating that it is difficult to distinguish different wear states by traditional linear interval division. Therefore, it is a nonlinear problem to identify three wear states by multifractal spectrum parameters of frictional vibration signals.

## 5. Wear state identification of frictional vibration signals with SVM method

### 5.1 Identification results under the same working condition

The samples under the loading force of 50 N and reciprocating frequency of 1 Hz were selected for analysis, and the labels of the samples under the three states of running-in wear, normal wear and severe wear were set as 1, 2 and 3 in turn. To avoid the weight of the trained model was too small, which will cause the instability of the numerical calculation, and make the parameter optimization converged at a faster speed, the data was normalized by using the mapminmax function. A total of 210 groups of 70 groups in each state were randomly selected as training samples, and the seven-dimensional normalized feature vector of the 210 groups of samples was obtained. A total of 90 groups of 30 groups in each state were randomly selected from the remaining sample set as test samples. The original SVM classification method is used to solve two classification problems. For the three classification problems in this paper, the one-to-one method [22] was adopted to identify three different wear states, radial basis kernel function [23] was adopted, and SVM parameters was optimized by grid optimization method [24].

**Fig 10:**
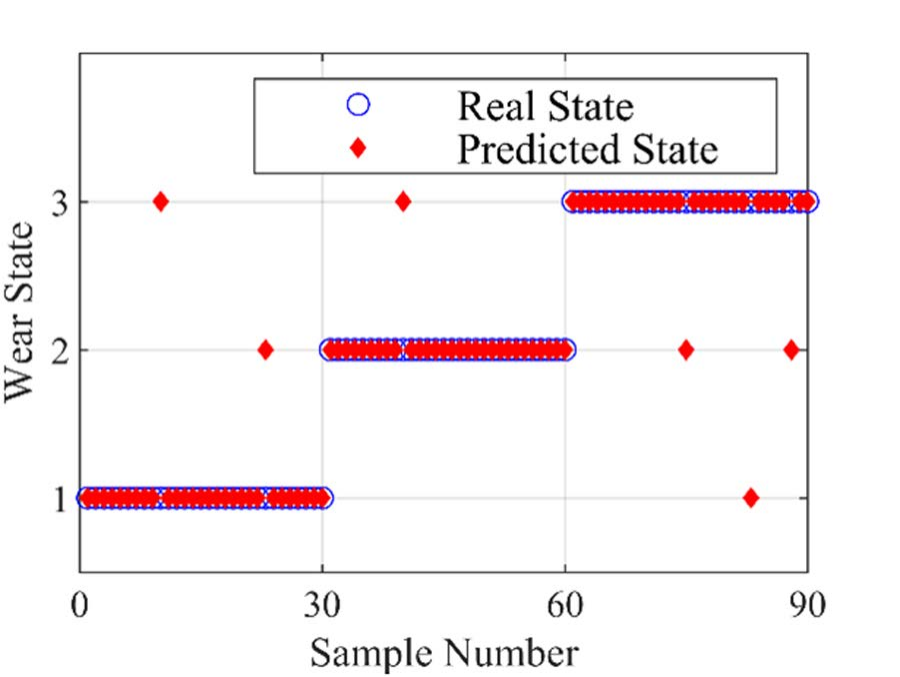
SVM recognition results with grid optimization.

The identification results are shown in Fig 10. As shown in Fig 10, only six samples are misjudged, and the overall recognition accuracy is 93.3%, indicating that three wear states can be identified by SVM. Table 3 shows the recognition accuracy of different wear states. The recognition accuracy of severe wear state is the lowest, while the recognition accuracy of normal wear state is the highest, which may be caused by the instability of severe wear state compared with normal wear state.

**Table 3:** Recognition accuracy of different wear states.

Wear state	Running-in	Normal	Severe	Accuracy (%)
Train set	70	70	70	95.7
Test set	30	30	30	93.3
Accuracy (%)	93.3	96.7	90	–

To rigorously address the request for comparative evaluation with alternative machine learning algorithms and comprehensive classification metrics, we conducted systematic benchmarking against Long short-term memory (LSTM), Gated Recurrent Unit(GRU), and Random Forest (RF). All models were trained and validated using identical experimental datasets—210 samples for training and 90 samples for testing—under controlled conditions (50N load, 1 Hz frequency). Performance was quantitatively assessed through a suite of metrics including confusion matrices, precision, recall, F1-score, and overall accuracy. As demonstrated in Table 4, the proposed nonlinear SVM classifier achieved superior performance, attaining peak accuracy (93.3%) and F1-score (0.932). These results robustly validate SVM’s enhanced capability in modeling complex nonlinear relationships inherent in multifractal feature spaces—a critical advantage over conventional algorithms for wear state identification.

**Table 4:** Comparison of accuracy of different methods.

Model	Accuracy (%)	Precision	Recall	F1-Score
SVM	93.3	0.932	0.933	0.932
LSTM	85.6	0.847	0.856	0.849
GRU	88.9	0.882	0.889	0.885
RF	80.0	0.786	0.800	0.791

To rigorously evaluate the generalization capability of the proposed nonlinear support vector machine (SVM) model and mitigate overfitting risks, a tenfold cross-validation procedure was implemented in accordance with established machine learning validation practices [25–27]. The dataset comprised 210 samples (70 per wear state: running-in, normal, and severe wear), randomly partitioned into 10 equally sized subsets. During each iteration, 9 subsets (189 samples) were used for training, while the remaining subset (21 samples) served as the validation set. This process repeated iteratively until all subsets were validated.

As summarized in Table 5, the model achieved a mean accuracy of 94.0% (standard deviation ±1.5%) across all validation rounds. The consistency of performance—ranging from 89.8% (Round 6) to 96.0% (Round 8)—confirms the method’s robustness to data partitioning variations. Notably, the severe wear state exhibited higher volatility (90.5% ± 3.3%), attributable to two key factors: Friction coefficient fluctuations under severe wear conditions; Feature aliasing in multifractal parameters, particularly in Δf and $\alpha_{\min}$. This variability aligns with transitional dynamics observed during wear-state transitions and underscores the challenge of distinguishing severe wear when multifractal parameters overlap with running-in states. Nevertheless, the sustained >90% accuracy across all states validates the SVM’s efficacy for wear-state identification.

**Table 5:** Comprehensive tenfold cross-validation performance metrics.

Round	Running-in (%)	Normal (%)	Severe (%)	Overall Accuracy (%)
1	95.2	100.0	85.7	93.3
2	90.5	95.2	90.5	92.1
3	94.3	97.6	88.1	93.3
4	92.9	100.0	90.5	94.5
5	95.2	97.6	92.9	95.2
6	88.1	95.2	86.0	89.8
7	94.3	97.6	92.9	94.9
8	95.2	100.0	92.9	96.0
9	90.5	95.2	88.1	91.3
10	93.0	97.6	95.2	95.3
Mean ± SD	93.8 ± 2.1	97.6 ± 1.9	90.5 ± 3.3	94.0 ± 1.5

### 5.2 Analysis of the influence of loading force on recognition accuracy

The SVM was trained by randomly selecting 50 samples of frictional vibration signals under various wear conditions with loading force of 70N with reciprocating frequency 0.5 Hz, which were generated by feature extraction of noise reduction processing. 50 samples of frictional vibration signals in each wear state under the loading force of 50 N with reciprocating frequency of 0.5 Hz, and the loading force of 60N with reciprocating frequency of 0.5 Hz were selected as test set samples for test analysis.

**Fig 11:**
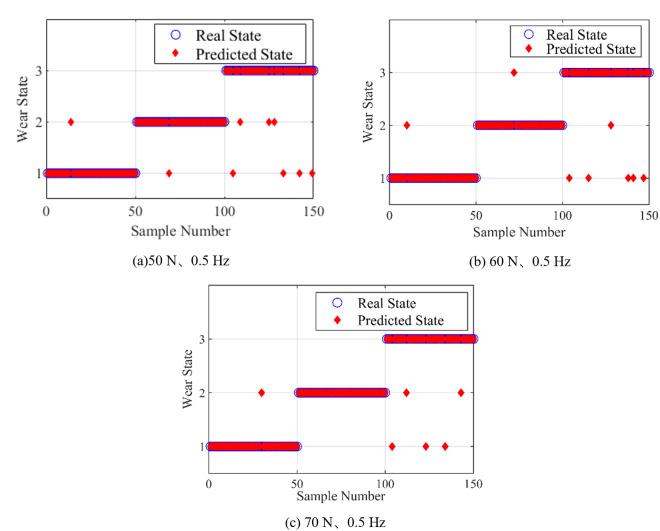
SVM recognition results under different loading force.

The recognition results are shown in Fig 11. As shown in Fig 11a, nine samples are misjudged with the loading force 50 N, and the recognition accuracy is 94%. As shown in Fig 11b, eight samples are misjudged with the loading force 60 N, and the recognition accuracy is 94.7%. six samples are misjudged with the loading force 70 N, and the accuracy of recognition is 96%. The sample recognition accuracy of the test set under different loading is similar to each other. Therefore, it can be concluded that the change of loading force has almost no effect on the model recognition results.

### 5.3 Analysis of the influence of reciprocating frequency on recognition

SVM was trained by randomly selecting 50 samples of frictional vibration signals in various wear states under conditions of 50 N loading force and 2 Hz reciprocating frequency, which were generated by feature extraction of noise reduction processing. The classification method was one-to-one, kernel function was radial basis kernel function, and parameter optimization method was grid optimization. 50 samples of frictional vibration signals in different wear states with reciprocating frequency of 0.5 Hz, 1 Hz and 2 Hz were selected for testing.

**Fig 12:**
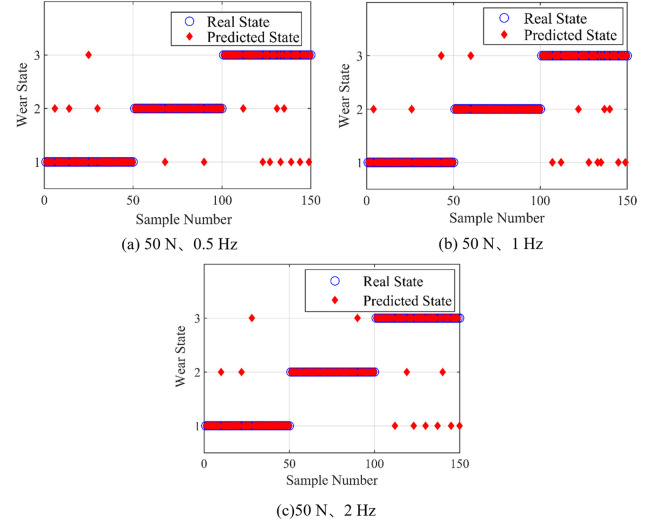
SVM recognition results under different reciprocating frequency.

The recognition results are shown in Fig 12. As shown in Fig 12a, fifteen samples are misjudged with reciprocating frequency 0.5 Hz, and the accuracy of recognition is 90%. As shown in Fig 12b, fourteen samples are misjudged with reciprocating frequency 1 Hz, and the accuracy of recognition is 90.7%. As shown in Fig 12c, twelve samples are misjudged with reciprocating frequency 2 Hz, and the accuracy of recognition is 92%. The sample recognition accuracy of the test set under different reciprocating frequency is similar to each other. Therefore, it can be concluded that the change of reciprocating frequency has almost no impact on the model recognition results.

## 6. Conclusion

This research proposes a novel method for identifying the wear state of reciprocating sliding friction pairs based on fractal parameters of frictional vibration signals and nonlinear support vector machines, addressing the challenge of real-time monitoring of wear states. Frictional vibration is an inevitable vibrational phenomenon generated during the relative motion of friction pairs, which can be conveniently collected in real-time engineering practice. The study revealed that frictional vibration signals exhibit distinct multifractal characteristics, and the distribution of multifractal spectrum parameters under different wear conditions demonstrates excellent nonlinear identifiability. Based on this finding, this paper proposes constructing feature vectors from the fractal parameters of frictional vibration signals and employing nonlinear support vector machines for pattern recognition and classification of different wear states. Through experimental research, the wear process was divided into three typical states: running-in wear, normal wear, and severe wear. The results demonstrated that the proposed method achieved recognition accuracies exceeding 90% for all three wear states, validating its effectiveness. This achievement not only provides a new technical approach for friction wear monitoring but also offers a valuable reference for the application of nonlinear signal processing in other fields.
